# The Impact of Cardiac Dysfunction Based on Killip Classification on Gastrointestinal Bleeding in Acute Myocardial Infarction

**DOI:** 10.3389/fmed.2022.865663

**Published:** 2022-06-23

**Authors:** Yu Liu, De-Jing Feng, Le-Feng Wang, Li-Hong Liu, Zheng-Hong Ren, Jian-Yu Hao, Kui-Bao Li, Mu-Lei Chen

**Affiliations:** ^1^Heart Center and Beijing Key Laboratory of Hypertension, Beijing Chaoyang Hospital, Capital Medical University, Beijing, China; ^2^School of Public Health, Peking University Health Science Center, Beijing, China; ^3^Department of Gastroenterology, Beijing Chaoyang Hospital, Capital Medical University, Beijing, China

**Keywords:** acute myocardial infarction, Killip classification, stress ulcer, gastrointestinal bleeding, cardiac function

## Abstract

**Background:**

Owing to limited data, the effect of cardiac dysfunction categorized according to the Killip classification on gastrointestinal bleeding (GIB) in patients with acute myocardial infarction (AMI) is unclear. The present study aimed to investigate the impact of cardiac dysfunction on GIB in patients with AMI and to determine if patients in the higher Killip classes are more prone to it.

**Methods:**

This retrospective study was comprised of patients with AMI who were admitted to the cardiac intensive care unit in the Heart Center of the Beijing Chaoyang Hospital between December 2010 and June 2019. The in-hospital clinical data of the patients were collected. Both GIB and cardiac function, according to the Killip classification system, were confirmed using the discharge diagnosis of the International Classification of Diseases, Tenth Revision coding system. Univariate and multivariate conditional logistic regression models were constructed to test the association between GIB and the four Killip cardiac function classes.

**Results:**

In total, 6,458 patients with AMI were analyzed, and GIB was diagnosed in 131 patients (2.03%). The multivariate logistic regression analysis showed that the risk of GIB was significantly correlated with the cardiac dysfunction [compared with the Killip class 1, Killip class 2’s odds ratio (*OR*) = 1.15, 95% confidence interval (*CI*): 0.73–1.08; Killip class 3’s *OR* = 2.63, 95% *CI*: 1.44–4.81; and Killip class 4’s *OR* = 4.33, 95% *CI*: 2.34–8.06].

**Conclusion:**

This study demonstrates that the degree of cardiac dysfunction in patients with acute myocardial infarction is closely linked with GIB. The higher Killip classes are associated with an increased risk of developing GIB.

## Introduction

Patients hospitalized with acute myocardial infarction (AMI) have an increased risk of gastrointestinal bleeding (GIB) because of the possibility of stress-related gastrointestinal mucosal erosion or ulcers and the need for antiplatelet and/or anticoagulation agents (1–3). This bleeding can aggravate and increase the severity of the primary disease, thereby increasing the risk of death (1, 4). The previous clinical studies have revealed that the incidence of stress ulcers (SUs) is related to the severity of the primary disease (5). Therefore, SUs are more likely to develop in patients with a more severe primary disease. AMI is a common clinical disease with different clinical severities in the acute phase, ranging from stable hemodynamic status to cardiogenic shock.

There is a paucity of data regarding the impact of different levels of cardiac dysfunction on GIB in patients with AMI. In the present study, it is hypothesized that the incidence of GIB is correlated with the level of cardiac dysfunction during AMI. This study aims to investigate the impact of cardiac dysfunction on GIB in patients with AMI and determine if patients in the higher Killip classes are more prone to GIB. It is believed to be one of only a small number of studies that investigate the association between every Killip class and GIB.

## Materials and Methods

### Study Population and Ethical Considerations

The database concerning consecutive adult patients with AMI who were ≥ 18 years of age and were admitted to the cardiac intensive care unit (CICU) of the Heart Center at the Beijing Chaoyang Hospital affiliated to the Capital Medical University between December 1, 2010, and June 31, 2019, was analyzed. Patients who died or were discharged before a definite assessment of their cardiac function and GIB were excluded, as were patients with GIB before the onset of AMI and those with esophageal variceal bleeding. In total, 6,458 patients were enrolled and 36 were excluded. Of the analyzed cases, 2,663 patients (41.2%) had a non-ST-elevation myocardial infarction (NSTEMI) and 3,795 (58.7%) had an ST-elevation myocardial infarction (STEMI).

The diagnostic criteria for AMI were as follows: the STEMI diagnosis was based on the European Heart Association 2017 STEMI diagnostic criteria, including chest pain with a new ST-segment elevation in more than two adjacent electrocardiogram leads or a new left bundle branch block (6), and the NSTEMI diagnosis was based on the 2015 diagnostic criteria, including chest pain and new onset of an ST-segment depression in more than two adjacent electrocardiogram leads, with an increase in serum troponin I after admission to more than three times the upper limit of the normal value (7).

Overt GI bleeding was defined as hematemesis (the vomiting of bright red blood or dark brown granular material that resembles coffee grounds) and hematochezia or melena). Clinically important GI bleeding was defined as overt bleeding complicated by one of the following within 24 h after the onset of bleeding (in the absence of other causes): a spontaneous decrease of more than 20 mmHg in the systolic blood pressure; an increase of 20 beats per min in the heart rate or a decrease of more than 10 mmHg in the systolic blood pressure measured on sitting up; a decrease in hemoglobin of at least 2 g/dl (1.24 mmol/L); or a subsequent transfusion of two or more units of red blood cells during the bleeding episode (5).

This retrospective clinical trial was conducted at Beijing Chaoyang Hospital affiliated with the Capital Medical University, and the ethics committee of the hospital approved the study (approval number: 2019-scientific-5-1). Due to the retrospective nature of the study, the requirement for informed consent was waived.

### Data Collection

All data were obtained from the Beijing Chaoyang Hospital, which is one of the most influential hospitals in China for the treatment of coronary heart disease. The Heart Center of the hospital established one of the first primary percutaneous coronary intervention teams in China in 1993 and is well-known for its quick response in performing reperfusion on eligible patients with AMI. The patients in the study are representative of the general Beijing population.

The electronic medical records were reviewed by the survey doctors (LY, FDJ, ZDP, ZZY, LN, and FYF), who collected the basic demographic information, previous medical history, admission diagnosis, therapeutic information, and discharge prescription(s) of all the patients. Diagnoses of digestive tract tumor history, peptic ulcer history, stroke, anxiety and depression status, and renal insufficiency were based on the International Classification of Diseases Tenth Revision (8) diagnostic information, which was provided by the Information Center of the Beijing Chaoyang Hospital. The codes of K25, K26, K27, and K92 were screened for. All GIB events were confirmed by independent gastroenterology consultant (HJY) after the onset of AMI and during hospitalization, and patients with preexisting GIB were excluded.

Diagnoses of hyperlipidemia, diabetes, and anemia were based on medical history combined with laboratory tests during admission. The smoking status and alcohol use were obtained from the electronic medical records. Non-steroidal anti-inflammatory drug (NSAID) history referred to the use of NSAIDs before admission, and a history of NSAID use was recorded if the patient had not stopped using them at least 3 months before admission. Antiplatelet drug history referred to the use of aspirin and/or a P2Y12 receptor antagonist (RA) within 3 months of admission. Aspirin and clopidogrel were administered in an oral dosage form of 100 and 75 mg, respectively, in all cases.

### Cardiac Function Classification

The cardiac function during the acute phase of AMI objectively reflects the severity of the AMI. The Killip classification method is simple and accurate and has been widely adopted by clinicians over the past five decades to assess the severity of an AMI. In this study, the Killip classification was used to determine the severity of the AMI and analyzed the relationship between the different cardiac function levels and GIB. The Killip classification was determined based on the previous literature as follows: class 1 (K1), no signs of heart failure; class 2 (K2), signs indicating mild to moderate heart failure (i.e., third heart sound gallop, rales halfway up the lung fields, or elevated jugular venous pressure); class 3 (K3), pulmonary edema; and class 4 (K4), cardiogenic shock or refractory hypotension (9).

Echocardiographic ejection fraction is a more quantitative and accurate method to evaluate the cardiac function than Killip classification. The latter is easier to implement without the help of medical equipment. In some cases, echocardiographic ejection fraction was collected, because not all patients were examined during CICU, or the data were lost. The two non-invasive methods to evaluate the cardiac function were compared in AMI episode.

### In-Hospital Medication

Information about each patient regarding the use of antithrombotic drugs and gastric mucosal protective agents during hospitalization was provided by the Pharmaceutical Center of the Beijing Chaoyang Hospital. The available gastric mucosal protective agents included: (1) proton pump inhibitors (PPIs), such as omeprazole, pantoprazole, rabeprazole, and esomeprazole; (2) Histamine-2 receptor antagonists (H2RAs), such as cimetidine, ranitidine, and famotidine; and (3) oral gefarnate. An intravenous PPI was the first choice for the prophylactic treatment of peptic ulcers. Intravenous H2RAs were given when patients were allergic to PPIs or had liver and kidney damage. The PPIs or H2RAs were used for 3–5 days during a CICU stay. Oral gefarnate (one to two tablets, three times a day) was administered once the intravenous prophylaxis was withdrawn, and the dose was maintained for 3 months.

### Definitions

In line with previously established criteria, anemia was diagnosed when the baseline hematocrit value was < 39% for men and < 36% for women (10). Likewise, the chronic renal insufficiency was defined as a creatinine clearance of < 60 ml/min (11), and thrombocytopenia was defined as a nadir in-hospital platelet count of < 100 × 10^9^/L (12).

### Endpoints

The endpoints were overt GIB and clinically important GIB. GIB event was performed by an independent gastroenterology consultant.

### Statistical Analysis

All statistical analyses were performed with SPSS Statistics 20.0 (IBM Company). The subjects were divided into four groups (Killip classes 1–4). Continuous variables with a normal distribution were expressed as the mean ± standard deviation (age and hospital stay); non-normally distributed variables were expressed as quartile intervals [median (Q1, Q3)], and countable data were expressed as frequency (*n*,%). According to the data type, either a one-way analysis of variance or a chi-squared test was used to compare the baseline characteristics between the groups. Univariate and multivariate conditional logistic regression models were constructed. The univariate logistic regression model was used to analyze the relationship between Killip classes 1–4 and GIB, and the multivariate regression model was used to adjust for common factors influencing GIB. All the variables were compared using a bilateral test, and *p* < 0.05 was statistically significant.

## Results

The data of 6,458 patients with AMI, with a mean age of 63.4 ± 12.8 years, were analyzed. All the patients were spent at least 24 h in the CICU, and gastric mucosal protective agents, i.e., PPIs, H2RAs, or gefarnate, were administered to them. GIB was confirmed in 131 patients. Eight of them had bleeding in the small bowel or lower gastrointestinal (GI) tract (2 in Killip class 1, 3 in Killip class 2, and 3 in Killip class 3), while all the rest had bleeding in the upper GI tract. Table 1 summarizes the baseline characteristics of the patients with and without GI bleeding. Patients who developed GI bleeding were older, more likely to be female, and tended to have a history of diabetes, dyslipidemia, renal insufficiency, anemia, or peptic ulcers. In addition, they had a lower left ventricular ejection fraction (46.08 ± 11.78 *vs*. 55.15 ± 13.04) at baseline and received more cardiopulmonary resuscitation. They had longer hospital stays and were more likely to have received treatment with PPIs, and they had a higher in-hospital death rate.

**TABLE 1:** Baseline characteristics of the study patients according to gastrointestinal bleeding (GIB) event.

	Without GI bleeding	With GI bleeding	*P*-value
	(*n* = 6327)	(*n* = 131)	
Age (year)	63.23 ± 12.79	69.08 ± 13.22	<0.001
<45	486 (7.68)	10 (7.63)	0.984
45–64	2,942 (46.50)	29 (22.14)	<0.001
65–79	2,170 (34.30)	65 (49.62)	<0.001
≥ 80	729 (11.52)	27 (20.61)	0.001
Female (%)	1,608 (25.41)	44 (33.59)	0.034
Alcohol use	1,520 (24.02)	26 (19.85)	0.268
Stroke (%) (%)	687 (10.86)	21 (16.03)	0.061
Use of NSAIDs	71 (1.12)	3 (2.29)	0.214
OAC user (%)	185 (2.92)	5 (3.82)	0.549
LVEF	55.15 ± 13.04	46.08 ± 11.78	<0.001
**Medical history**			
Hypertension, (%)	3,907 (61.75)	82 (62.60)	0.844
Hyperlipidemia	4,992 (78.90)	79 (60.31)	<0.001
Diabetes	2,144 (33.89)	58 (44.27)	0.013
Smoking	3,397 (53.69)	62 (47.33)	0.148
Alcohol use	2,505 (39.59)	41 (31.30)	0.055
Renal insufficiency	498 (7.87)	33 (25.19)	<0.001
Prior peptic ulcer	213 (3.37)	25 (19.08)	<0.001
Prior peptic tumor	70 (1.11)	2 (1.53)	0.650
Anemia	295 (4.66)	41 (31.30)	<0.001
Thrombocytopenia	39 (0.62)	0 (0)	0.367
Long time use of antiplatelet	652 (10.31)	12 (9.16)	0.669
**Medical procedure after onset of AMI**			
STEMI	3,732 (58.99)	63 (48.09)	0.012
Anterior wall	1,664 (26.30)	25 (19.08)	0.063
Cardiopulmonary resuscitation	168 (2.66)	11 (8.40)	<0.001
Anxiety and depression	39 (0.62)	1 (0.76)	0.832
Hospital stays (days)	10.45 ± 8.14	16.37 ± 12.35	<0.001
**Anticoagulant**			
Heparin or LMWH	6,327 (100)	131 (100)	NS
**Antiplatelet**			
Aspirin	6,202 (98.02)	126 (96.18)	0.138
P2Y12 receptor antagonist	6,131 (96.90)	125 (95.42)	0.335
GPIIb/IIIa receptor antagonist	3,440 (54.37)	62 (47.33)	0.109
**Protective agent for gastric mucosa**			
PPI	6,063 (95.83)	131 (100)	0.017
H_2_RAs	207 (3.27)	5 (3.82)	0.729
Gefarnate	5,323 (84.13)	114 (87.02)	0.369
In-hospital death	225 (3.56)	15 (11.45)	<0.001

*Values are n (%) unless otherwise indicated. The normal distribution data (age, LVEF and length of stay) are expressed by X ± s; the rest are counting data, expressed by frequency (percentage, %).*

*LVEF, left ventricular ejection fraction; AMI, acute myocardial infarction; GIB, gastrointestinal bleeding; GPIIb/IIIa, glycoprotein IIb/IIIa; H2RA, H2 receptor antagonists; LMWH, low–molecular-weight heparin; NSAIDs, non-steroidal anti-inflammatory drugs; NSTEMI, non-ST-segment elevation myocardial infarction; OAC, oral anticoagulation; PPI, proton pump inhibitor; SD, standard deviation; STEMI, ST-segment elevation myocardial infarction; SU, stress ulcer.*

### Endpoint Events

With respect to the 6,458 patients, the occurrence of GIB in Killip classes 1–4 was as follows: 42 out of 3,292 (1.27%) in the Killip class 1 group; 41 out of 2,372 (1.73%) in the Killip class 2 group; 26 out of 513 (5.07%) in the Killip class 3 group; and 22 out of 281 (7.83%) in the Killip class 4 group. The time of GIB from AMI onset (days) was shorter in the Killip class 1 group than in any other Killip class group, and the times of the other groups were comparable (Table 2).

**TABLE 2:** Patient characteristics and GIB in groups 1–4.

Variable	Killip classification				*P*-value
Case number	1 (*n* = 3292)	2 (*n* = 2372)	3 (*n* = 513)	4 (*n* = 281)	
Age (years)	59.57 ± 12.22	65.93 ± 12.28	71.86 ± 10.64	70.26 ± 12.28	<0.001
Female	704 (21.39)	665 (28.03)	189 (36.84)	94 (33.45)	<0.001
Hypertension	1,977 (60.05)	1,506 (63.49)	349 (68.03)	157 (55.87)	0.076
Diabetes	987 (29.98)	855 (36.05)	268 (52.24)	92 (32.74)	<0.001
Hyperlipidemia	2,731 (82.95)	1,869 (78.79)	336 (65.49)	135 (48.04)	<0.001
Stroke	284 (8.63)	299 (12.61)	87 (16.96)	38 (13.52)	<0.001
Smoking	1,825 (55.44)	1,246 (52.53)	249 (48.54)	139 (49.47)	0.005
Alcohol	1,352 (41.07)	912 (38.45)	184 (35.87)	98 (34.88)	0.021
Use of NSAIDs	25 (0.76)	30 (1.26)	12 (2.34)	7 (2.49)	<0.001
Use of OAC	83(2.52)	78 (3.29)	21 (4.09)	8 (2.85)	0.144
Renal insufficiency	116 (3.52)	207 (8.73)	133 (25.92)	75 (26.69)	<0.001
Peptic ulcer	132 (4.01)	85 (3.58)	16 (3.12)	5 (1.78)	0.046
Peptic tumor	32 (0.97)	27 (1.14)	9 (1.75)	4 (1.42)	0.152
Anemia	106 (3.22)	135 (5.69)	71 (13.84)	24 (8.54)	<0.001
Thrombocytopenia	17 (0.52)	14 (0.59)	5 (0.97)	5 (1.06)	0.671
Long time use of antiplatelet	340 (10.33)	238 (10.03)	60 (11.69)	26 (9.25)	0.995
NSTEMI	1,471 (44.68)	854 (36.00)	243 (47.37)	95 (33.81)	<0.001
Cardiopulmonary resuscitation	31 (0.94)	68 (2.87)	24 (4.68)	56 (19.93)	<0.001
Anxiety and depression	23 (0.69)	17 (0.72)	3 (0.58)	4 (1.42)	0.452
NSTEMI	1,471 (44.68)	854 (36.00)	243 (47.37)	95 (33.91)	<0.001
Ejection fraction	61.06 ± 10.94	58.66 ± 11.70	48.14 ± 11.03	46.94 ± 12.37	<0.001
Length of CICU stay (days)	2.14 ± 1.71	3.88 ± 2.32	6.96 ± 5.18	6.25 ± 6.51	<0.001
In-hospital death	31 (0.94)	53 (2.23)	53 (10.53)	105 (37.37)	<0.001
GIB	42 (1.28)	41 (1.73)	26 (5.07)	22 (7.83)	<0.001
Time of GIB from AMI onset (days)	2.14 ± 1.48	3.92 ± 3.35	4.81 ± 1.89	3.27 ± 2.24	<0.001
In-hospital use of antiplatelet	3,285 (99.79)	2,368 (99.83)	509 (99.22)	279 (99.29)	NS
Protective agent for gastric mucosa	3,292 (100)	2,372 (100)	513 (100)	281 (100)	NS

*The normal distribution data (age, length of ICU stay) are expressed by X ± s; the rest are counting data, expressed by frequency (percentage, %). AMI, acute myocardial infarction; GIB, gastrointestinal bleeding; CICU, cardiac intensive care unit; NSTEMI, non-ST-segment elevation myocardial infarction; SD, standard deviation.*

### The Association Between the Echocardiographic Ejection Fraction and Killip Classification and Gastrointestinal Bleeding

**FIGURE 1:**
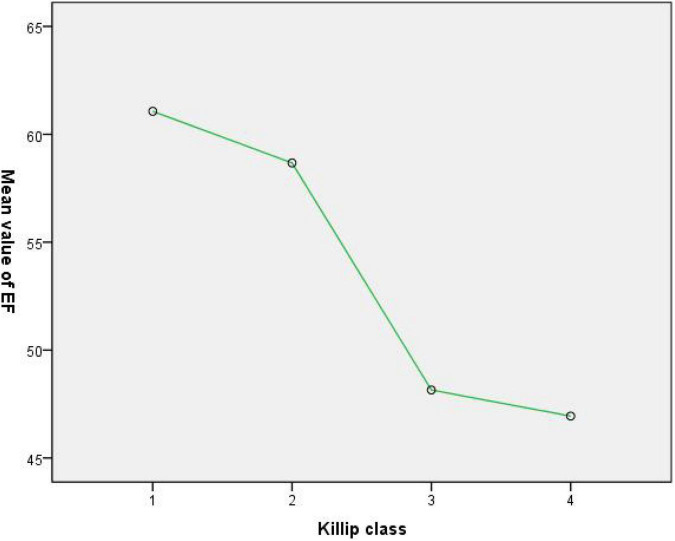
Higher Killip classes had lower echocardiographic ejection fraction.

There was a trend that the higher Killip classes had lower echocardiographic ejection fraction; and a significant difference between the ejection fraction of groups K2 and K3 (58.66 ± 11.70 vs. 48.14 ± 11.03, *p* < 0.001). With respect to groups K1 and K2, the ejection fraction was comparable (61.06 ± 10.94 vs. 58.66 ± 11.70, *p* = 0.054), as it was also comparable for groups K3 and K4 (48.14 ± 11.03 vs. 46.94 ± 12.37, *p* = 0.052) (see Table 2 and Figure 1).

**FIGURE 2:**
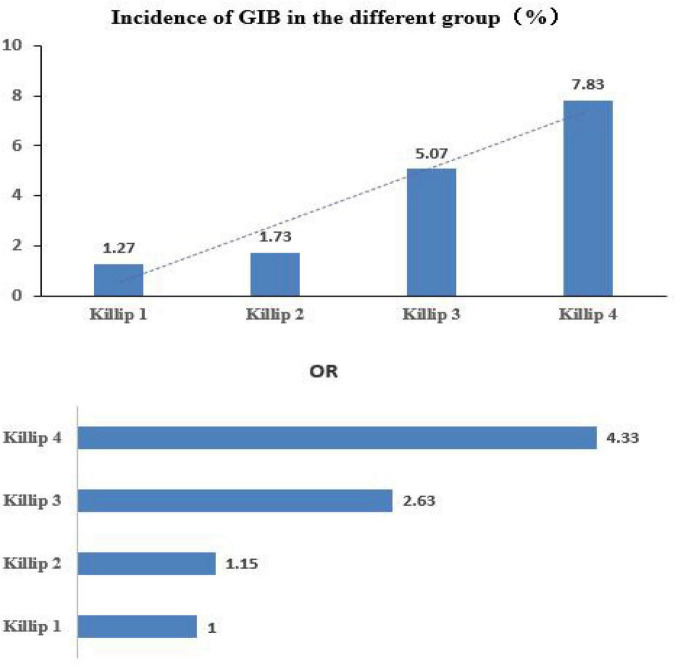
Higher Killip classes are associated with an increased risk of developing gastrointestinal bleeding.

The logistic regression analysis was performed using GIB as the dependent variable and the Killip classification as the independent variable. There was no significant difference between the class 2 and class 1 groups in the risk of GIB [odds ratio (*OR*) = 1.36, 95% confidence interval (*CI*): 0.88–2.09, and *p* = 0.164]. The risk in the class 3 group was 4.13 times higher than in the class 1 group (*OR* = 4.13, 95% *CI*: 2.51–6.79, and *p* < 0.001), and the risk in the class 4 group was 6.57 times higher than it was in the class 1 group (*OR* = 6.57, 95% *CI*: 3.86–11.18, and *p* < 0.001) (see Table 2 and Figure 2). After adjustment, the results show that the influence of the different Killip class levels of cardiac function on the occurrence of GIB remains significant (see Table 3).

**TABLE 3:** The adjusted relationship between Killip classification and GIB.

	OR			
Stepwise logistic regression	Killip 2	Killip 3	Killip 4	*P*-value
0	1.36 (0.88–2.09)	4.13 (2.51–6.79)	6.577 (3.86–11.18)	<0.001
1	1.21 (0.78–1.89)	3.28 (1.94–5.55)	5.36 (3.08–9.31)	<0.001
2	1.21 (0.78–1.89)	3.26 (1.93–5.53)	5.36 (3.08–9.31)	<0.001
3	1.22 (0.78–1.91)	3.33 (1.97–8.03)	5.29 (3.05–9.21)	<0.001
4	1.21 (0.77–1.88)	3.12 (1.84–5.32)	5.22 (2.99–9.09)	<0.001
5	1.19 (0.76–1.86)	3.11 (1.83–5.29)	5.09 (2.92–8.88)	<0.001
6	1.17 (0.75–1.83)	2.87 (1.66–4.98)	4.59 (2.56–8.22)	<0.001
7	1.22 (0.78–1.91)	3.23 (1.84–5.69)	4.78 (2.67–8.57)	<0.001
8	1.25 (0.80–1.96)	3.45 (1.96–6.09)	5.13 (2.85–9.24)	<0.001
9	1.25 (0.79–1.96)	3.44 (1.95–6.08)	5.12 (2.84–9.22)	<0.001
10	1.25 (0.79–1.95)	3.37 (1.89–5.98)	5.15 (2.86–9.27)	<0.001
11	1.23 (0.79–1.92)	3.23 (1.80–5.79)	5.06 (2.79–9.14)	<0.001
12	1.23 (0.79–1.93)	3.23 (1.80–5.79)	5.06 (2.75–9.33)	<0.001
13	1.15 (0.73–1.80)	2.63 (1.44–4.81)	4.33 (2.34–8.03)	<0.001
14	1.15 (0.73–1.80)	2.63 (1.44–4.81)	4.33 (2.34–8.06)	<0.001

*Odds ratio (OR) expressing the excess of risk of GIB. GI, gastrointestinal bleeding (GIB). Covariates were introduced into a logistic regression model stepwise. Covariant: 0 = Killip classification; 1 = 0 + age; 2 = 1 + sex; 3 = 2 + hypertension; 4 = 3 + diabetes; 5 = 4 + stroke; 6 = 5 + renal insufficiency; 7 = 6 + smoking and alcohol use; 8 = 7 + peptic ulcer; 9 = 8 + peptic tumor; 10 = 9 + anemia + thrombocytopenia; 11 = 10 + long time use of antiplatelet and NSAIDs + OAC; 12 = 11 + Cardiopulmonary resuscitation; 13 = 12 + Anxiety and depression + length of CICU stay; 14 = 13 + In-hospital use of antiplatelet and protective agent for gastric mucosa. CICU, cardiac intensive care unit; OAC, oral anticoagulation; OR, odds ratio; NSAIDs, non-steroidal anti-inflammatory drugs.*

## Discussion

This is a non-randomized comparative study, originating from the long-term clinical observation in our daily practice. GIB was found more likely to occur in AMI patients with worse cardiac dysfunction. We collected data to verify whether the two indexes are related. Echocardiographic assessment and Killip classification are two most used non-invasive methods to evaluate the cardiac function for patients in AMI episode. The former is quantitative and accurate while the Killip classification has been widely used without the help of complex medical equipment by clinicians over the past 50 years. Our study demonstrated the trend that the higher the Killip classes had lower echocardiographic EF values exists. In terms of evaluation of cardiac dysfunction, the two methods are consistent and have no conflict. The outstanding advantage of Killip classification is that it is simple and easy to operate without the help of complex medical equipment, which makes it easier to be operated by more clinicians and paramedics. In ambulances and some clinics with poor facilities, the cardiac function could be evaluated as early as possible. Hence, we adopted Killip classification to verify the relation between GIB and the cardiac function in patients presented with AMI in CICU.

### Acute Myocardial Infarction and Gastrointestinal Bleeding

The gastric (and sometimes the esophageal or duodenal) mucosal barrier is disrupted by the acute illness, and this disruption may present in the form of erosive gastritis ranging from asymptomatic superficial lesions and occult GI bleeding to overt clinically significant GIB (6, 13). However, although critically ill patients are often at risk of SUs, it has been discovered that not all patients with AMI develop them (5). The previous clinical studies have shown that the incidence of SUs is related to the severity of the primary disease (5).

Acute myocardial infarction is considered to be one of the common etiologies for the development of stress ulcers (such as gastritis and gastropathy), with less being intensively stressful than burns and cranial trauma (7, 9). On the other hand, the use of anticoagulant and dual antiplatelet therapy (DAPT) were recommended when treating patients with AMI (6). For these reasons, patients with AMI treated in CICU have an increased risk of GIB.

The 2017 ESC STEMI guideline recommended that a PPI in combination with DAPT is recommended in patients at high risk of GIB (6). It means prophylactic use of PPI is reasonable for high-risk bleeding patients and might not be necessary for those at low risk without pre-evaluation. Severe cardiac insufficiency in AMI episode increases the intensity of stress and gastrointestinal congestion. When considering the risk of GIB, the patient’s cardiac function status cannot be ignored.

The CRUSADE and DAPT score systems are recommended to identify the high-risk bleeding patients (14, 15). Both scoring systems identified the heart failure as a high risk factor for bleeding. Yet the degree of cardiac dysfunction during AMI scenario varies from asymptomatic to cardiogenic shock. Therefore, in the present study, it was hypothesized that different degrees of cardiac dysfunction have different contribution on GIB.

### The Level of Cardiac Function in Acute Myocardial Infarction and Gastrointestinal Bleeding

The cardiac dysfunction was already thought to be linked to GIB (16, 17). The present study found that the occurrence of GIB is significantly correlated, respectively, with each Killip class in patients with AMI.

Echocardiography was performed in 67.7, 79.8, 84.1, and 85.9% of the patients in the K1, K2, K3, and K4 groups, respectively. Meanwhile, Killip class was obtained for all cases. Since our study has demonstrated the relationship that the higher Killip classes had lower the echocardiographic EF values, the consistent assessment of cardiac function by two methods makes it credible to use Killip classification to evaluate the cardiac function.

The logistic regression model, after adjusting for age, sex, comorbidities, the use of protective agents for the gastric mucosa, and the use of antithrombotic drugs, showed that each Killip class has a different effect on GIB, and, thus, the classification can help to determine the risk of GIB in patients with AMI.

It is known that, in the acute phase of AMI, the location and duration of the occlusion of the culprit vessel, the infarct size, and the baseline characteristics lead to varying complications and result in different grades of hemodynamic disorders. Mild cases of AMI may be in a stable state throughout the acute phase, with fewer complications, and result in a shorter stay in the CICU. However, advanced cases may experience heart failure, hypotension, cardiopulmonary resuscitation, cardiogenic shock, multiorgan dysfunction, with more in-hospital complications, and higher mortality (18, 19).

The putative mechanisms include impaired cardiac function complicating low cardiac output leading to hypotension and subsequent gastrointestinal wall hypoxia, reducing gastric blood flow, and mucosal ischemia. The right-side cardiac dysfunction leads to the gastrointestinal wall congestion and hypoxia. Severe cardiac dysfunction aggravates the process and elevates the stress. The above pathological processes will lead to GIB.

### Endoscopy in the Acute Phase of Acute Myocardial Infarction

Endoscopic studies have shown that gastric erosions are present in up to 90% of patients in the intensive care unit (20, 21). In most patients, these lesions are superficial and asymptomatic. However, clinically important bleeding occurs in approximately 1% to 4% of critically ill patients (22). The previous studies have suggested that the endoscopy after AMI increases complications, such as hypotension, arrhythmia, and repeated acute coronary syndrome (23–26). This seems to be confirmed by the concerns of medical staff regarding this invasive examination during the acute phase of AMI and could explain why only two of the patients in this study, who had an urgent need, underwent endoscopy. Nevertheless, it is believed that this study is meaningful, as it provides a basis for clinicians to judge the risk of GIB according to the level of heart function of patients with AMI.

### Stress Ulcer Prophylaxis in Acute Myocardial Infarction

This study found that the stress ulcer prophylaxis (SUP) had been given to all the AMI patients at admission and during hospitalization to prevent GIB. In 2008, the American College of Cardiology and the American Heart Association, in association with the American College of Gastroenterology recommends gastroprotection with a PPI for all patients receiving DAPT (27). The use of SUP has also been suggested for the prevention and treatment of SUs in critically ill patients, with cardio-cerebral accident listed as a potential stressor (28, 29). Yet in a recent meta-analysis, Wang et al. (30) suggested that SUP reduced clinically important GIB when compared with a placebo or no prophylaxis in all-cause critical patients with a high risk of developing GIB, but the reduction in bleeding might be unimportant for low-risk patients. Thus, it is reasonable to use SUP according to the risk stratification of GIB in patients with acute myocardial infarction.

The results of the current study provide another consideration for clinicians and medical practitioners to decide the risk of GIB in patients with AMI episode. Patients with higher Killip classes are more likely to develop GIB when other bleeding risk factors are the same.

This study does not consider death as an endpoint because it is focused on the association between the degree of cardiac dysfunction and GIB, which, it is believed, has not been investigated before.

### Limitations

This study has some limitations that should be addressed. Being a retrospective observational study, this study may have inherent shortcomings. It is less convincing than a perspective one and we hypothesized that randomized control clinical trials may be needed to test if prophylactic use of PPI is unnecessary in AMI patients with normal cardiac function and without high-risk bleeding conditions. Second, none of the patient in our study underwent GI endoscopy, thus the exact site of bleeding remained unknown and the endoscopic incidence of SUs in patients in different Killip classes was not investigated.

## Conclusion

In conclusion, the degree of cardiac dysfunction in patients with acute myocardial infarction is closely linked with GIB and the higher Killip classes are associated with an increased risk of GIB.

## Data Availability Statement

The original contributions presented in the study are included in the article/supplementary material, further inquiries can be directed to the corresponding author.

## Ethics Statement

The studies involving human participants were reviewed and approved by the Beijing Chaoyang Hospital affiliated to the Capital Medical University (approval number: 2019-scientific-5-1). Written informed consent for participation was not required for this study in accordance with the national legislation and the institutional requirements.

## Author Contributions

YL and L-FW: conception, design of the research, and obtaining financing. YL and D-JF: acquisition of data. L-FW, L-HL, and J-YH: analysis and interpretation of the data. Z-HR and K-BL: statistical analysis. YL: writing of the manuscript. M-LC and J-YH: critical revision of the manuscript for intellectual content. All authors read and approved the final draft.

## Conflict of Interest

The authors declare that the research was conducted in the absence of any commercial or financial relationships that could be construed as a potential conflict of interest.

## Publisher’s Note

All claims expressed in this article are solely those of the authors and do not necessarily represent those of their affiliated organizations, or those of the publisher, the editors and the reviewers. Any product that may be evaluated in this article, or claim that may be made by its manufacturer, is not guaranteed or endorsed by the publisher.
